# Influence of Preexisting Psychiatric Morbidity on Liothyronine Use in Hypothyroidism: A Swedish Nationwide Cohort Study

**DOI:** 10.1210/clinem/dgaf337

**Published:** 2025-06-07

**Authors:** Fredric Hedberg, Jonatan D Lindh, Buster Mannheimer, Tereza Planck, Jakob Skov, Mikael Lehtihet, Henrik Falhammar, Jan Calissendorff

**Affiliations:** Department of Molecular Medicine and Surgery, Karolinska Institutet, Karolinska University Hospital L1:00, Stockholm SE-171 76, Sweden; Department of Endocrinology, Karolinska University Hospital, Stockholm 17176, Sweden; Department of Laboratory Medicine, Division of Clinical Pharmacology, Karolinska Institutet, Stockholm 14152, Sweden; Department of Clinical Science and Education at Södersjukhuset, Karolinska Institutet, Stockholm 11883, Sweden; Department of Internal Medicine, Section Diabetes and Endocrinology, Södersjukhuset, Stockholm 11883, Sweden; Department of Endocrinology, Skåne University Hospital, Malmö 21428, Sweden; Department of Clinical Sciences Malmö, Lund University, Lund 22184, Sweden; Department of Molecular Medicine and Surgery, Karolinska Institutet, Stockholm 17176, Sweden; Department of Medicine, Karlstad Central Hospital, Karlstad 65185, Sweden; Department of Medicine, Huddinge, Karolinska Institutet, Stockholm 14186, Sweden; Department of Endocrinology, Karolinska University Hospital, Stockholm 17176, Sweden; Department of Molecular Medicine and Surgery, Karolinska Institutet, Stockholm 17176, Sweden; Department of Endocrinology, Karolinska University Hospital, Stockholm 17176, Sweden; Department of Molecular Medicine and Surgery, Karolinska Institutet, Stockholm 17176, Sweden

**Keywords:** LT3, autoimmune hypothyroidism, combination therapy, psychiatric morbidity, epidemiology

## Abstract

**Context:**

Autoimmune hypothyroidism is a common endocrine disorder affecting 1% to 2% of the population in iodine sufficient areas. While levothyroxine is standard treatment, a substantial number of patients report persistent symptoms despite adequate treatment. The use of liothyronine as an adjunct to levothyroxine therapy has increased. The psychiatric characteristics of patients receiving liothyronine remain largely unknown.

**Objective:**

This study examines the association between preexisting psychiatric morbidity and subsequent liothyronine use in autoimmune hypothyroidism.

**Methods:**

This nationwide retrospective cohort study includes all adults in Sweden with autoimmune hypothyroidism and initiated on treatment with thyroid hormones between 2006 and 2020. Data were obtained from the National Patient Register and the National Prescribed Drug Register. Psychiatric morbidity prior to diagnosis was identified using ICD-10 codes and ATC codes for psychiatric medications. Logistic models estimated associations, adjusting for sex, age, and region.

**Results:**

Among 353 708 patients, 44.8% had a history of psychiatric morbidity. These patients were more likely to receive liothyronine (adjusted odds ratio [aOR] 1.90, 95% CI 1.83-1.97, *P* < .001) than those without a psychiatric history. This was most evident among individuals with affective or anxiety morbidity (aOR 1.91, 95% CI 1.84-1.98, *P* < .001). No association was found for psychotic morbidity (aOR 1.08, 95% CI 0.98-1.19, *P* = .11).

**Conclusion:**

Patients with a psychiatric history before autoimmune hypothyroidism were more likely to receive liothyronine, especially among those with affective or anxiety morbidity. This may reflect persistent symptoms and affect subsequent decisions in the treatment of hypothyroidism.

Hypothyroidism is one of the most common endocrine disorders, affecting 1% to 2% of the population in iodine-replete areas (1). Historically, hypothyroidism and myxedema were treated with various methods such as thyroid transplants and thyroid tissue preparations administered orally or via hypodermic injections (2). Modern treatments have since evolved to synthetic levothyroxine (LT4), which today remains the standard therapy (3).

While LT4 effectively normalizes thyroid hormone concentrations in most patients, a substantial minority of up to 10% continue to report persistent symptoms of hypothyroidism despite achieving biochemical euthyroidism (4). The reasons for these unresolved symptoms are not fully understood, and factors such as low intracellular triiodothyronine concentrations, autoimmunity, and comorbid conditions have been suggested (4).

In recent years, the use of liothyronine (LT3) as an add-on to LT4 therapy has increased (5, 6). By 2023 Sweden had 12 667 individual LT3 users, an 833% increase since 2006 (7). The increased use of LT3 highlights the needs for more personalized treatment approaches. Although some evidence suggests potential advantages in mental health, well-being, and patient preference, recent systematic reviews and meta-analyses from 2019, 2021, 2022, and 2024 (8-11) found no significant clinical benefits of combination therapy over LT4 monotherapy.

Current European guidelines support LT3 as an experimental add-on treatment in selected cases, but concerns about potential adverse effects on long-term cardiovascular health and bone metabolism have limited its widespread acceptance (12).

Despite the growing use of LT3, little is known about the characteristics of patients who receive LT3 and there is limited research on whether such patients exhibit distinct patterns of psychiatric morbidity or comorbid conditions prior to the diagnosis hypothyroidism. In clinical practice, we have noted that psychological distress seems to be increased in individuals who request LT3, but we have not found any published evidence. Understanding this patient subset is critical for evaluating the clinical context in which LT3 is prescribed and for assessing its potential risks and benefits.

This study aims to address this gap by investigating psychiatric comorbidities before the diagnosis of autoimmune hypothyroidism and later initiation of LT3 treatment.

## Materials and Methods

### Ethics Approval

This study was performed in line with the principles of the Declaration of Helsinki. The Swedish Ethical Review Authority approved the study. Informed consent was not required due to the retrospective nature of the study.

### Data Sources and Study Population

This retrospective, population-based cohort study covering the entire Swedish population, used data from 2 national registers maintained by the Swedish National Board of Health and Welfare: the National Prescribed Drug Register (NPDR) and the National Patient Register, the first covering all prescribed medications and the latter covering all inpatient as well as all specialist outpatient care. The study population included all individuals aged 18 years or older residing in Sweden who had at least 1 dispensation of thyroid hormone substitution, identified using Anatomical Therapeutic Chemical (ATC) code H03A between January 1, 2005, and December 31, 2020. Data were linked at the individual level using the unique 10-digit personal identity number assigned to all residents in Sweden (13).

The NPDR contains information on all prescribed drugs dispensed at pharmacies across Sweden from July 2005 and onwards (14). From this register, data on age, sex, county of residence, and medication use were extracted. Patients with at least 1 prescription of LT4 or LT3 were classified as LT4 or LT3 users, respectively.

The National Patient Register, encompassing both inpatient and outpatient specialist care, was used to identify diagnoses coded according to the International Classification of Diseases, according to the Swedish version of the Tenth Revision (ICD-10) since 1997. The register has a near complete coverage and a positive predictive value on diagnoses between 85% and 95% (15). Data on hospitalizations have been available since 1987, and outpatient visits in specialist care have been recorded since 2001. Patients with known causes other than autoimmune hypothyroidism, ICD-10 codes E030, E031, E050, C73, E230-E237, and E890, between 1997 and 2020, were excluded from the study (Table 1). Additionally, patients treated with amiodarone (ATC code C01BD01) or lithium (ATC code N05AN01) between 2005 and 2020 were also excluded (Table 1).

**Table 1.** ICD-10 and ATC codes with their definitions used in the study

Category	Codes	description	Excluded codes	Details of excluded codes
Thyroid hormone medications	ATC: H03A	H03A: thyroid hormones, H03AA01: levothyroxine, H03AA02: liothyronine, H03AA03: levothyroxine + liothyronine, H03AA04: tiratricol*^a^*, H03AA05: desiccated thyroid extract*^a^*	—	—
Psychiatric morbidity	ICD-10: F04-F99	Includes all psychiatric disorders	ICD-10 codes: F05.1, F70, F80	F05.1: delirium due to dementia, F70: intellectual disabilities, F80: developmental speech/language disorders
	ATC: N05, N06A	Includes psychiatric medications	N05AB01, N05AB04, N06AA	N05AB01: dixyrazine, N05AB04: prochlorperazine, N06AA: nonselective monoamine reuptake inhibitors (tricyclic antidepressants)
Affective/anxiety morbidity	ICD-10: F30-F48	Affective and anxiety disorders	—	—
	ATC: N05AN, N05B, N05C, N06A	Medications used for affective/anxiety disorders	N06AA	N06AA: Nonselective monoamine reuptake inhibitors (tricyclic antidepressants)
Psychotic morbidity	ICD-10: F20-F29	Psychotic disorders	—	—
	ATC: N05A	Medications used for psychotic disorders	ATC: N05AA02, N05AB01, N05AB04, N05AN	N05AA02: levomepromazine, N05AB01: dixyrazine, N05AB04: prochlorperazine, N05AN: lithium
Exclusion—other causes of hypothyroidism	ICD-10: E030, E031, E050, C73, E230-E237, E890	Other known causes of hypothyroidism	ICD-10: E030, E031, E050, C73, E230-E237, E890	E030-E031: congenital hypothyroidism, E050: Graves disease, C73: thyroid cancer, E230-E237: pituitary dysfunction (eg, central hypothyroidism), E890: postsurgical and radioiodine-induced hypothyroidism
Exclusion—medication use	ATC: C01BD01, N05AN01	medication affecting thyroid function	ATC: C01BD01, N05AN01	C01BD01: amiodarone, N05AN01: lithium

Abbreviations: ATC, Anatomical Therapeutic Chemical; ICD, International Classification of Diseases.

^*a*^Not registered in Sweden, available only by special license.

Index was the prescription date for the first dispensation of thyroid hormone, regardless of type (H03A). Patients with any dispensation of thyroid hormone with prescription date prior to July 1, 2006, were excluded to ensure that only newly treated patients were included. Each patient was followed from index until the occurrence of the outcome or until the end of the study period.

### Exposures and Outcomes

The primary exposure was psychiatric morbidity from the introduction of ICD-10 in 1997 to just before the individual index date, defined by ICD-10 codes F05-F99 (excluding F70, F80, and F05.1) and/or psychiatric medications identified with ATC codes N05 and N06A (excluding N05AB01, N05AB04, and N06AA) (Table 1). Separate secondary analyses evaluated specific psychiatric subcategories: affective or anxiety morbidity (ICD-10 codes F30-F48 and/or ATC codes N05AN, N05B, N05C, and N06A excluding N06AA) and psychotic morbidity (ICD-10 codes F20-F29 and/or ATC codes N05A excluding N05AA02, N05AB01, N05AB04, and N05AN) (Table 1).

The outcome was defined as dispensation of LT3 (ATC codes H03AA02 and H03AA03) at any time during the study period.

### Statistical Analyses

Descriptive data were summarized as medians and interquartile ranges (IQR). Categorical variables were reported as frequencies (n) and percentages (%). The chi square test was used to compare categorical variables, and the nonparametric Mann–Whitney U-test for continuous variables.

Logistic regression models were used to estimate the association between psychiatric morbidity prior to the diagnosis of autoimmune hypothyroidism and later prescription of LT3. Separate models were performed for the primary analysis (psychiatric morbidity) and secondary analyses (affective or anxiety morbidity and psychotic morbidity). The dependent variable was defined as dispensation of LT3, while the independent variables were psychiatric morbidity, affective or anxiety morbidity, and psychotic morbidity.

All analyses were adjusted for potential confounders, including age (categorized as 18-29, 30-49, 50-74, and 75+ years), sex, and county of residence at index date. In the primary analysis, separate logistic regression models were constructed for each confounder to assess their individual contribution to the adjusted odds ratio (aOR). A sensitivity analysis stratified by the index periods 2005-2011 and 2012-2020 was conducted to account for the temporal trend of increased prescribing patterns of LT3 after 2012 (7).

Results were reported as odds ratios (OR) and aOR with 95% CI. Statistical significance was set at *P* < .05.

All analyses were performed in R software (version 4.3.1), including packages data.table and survival.

## Results

### Baseline Characteristics

The study population consisted of 353 708 patients treated with thyroid hormones for autoimmune hypothyroidism. In the total cohort, 158 468 (44.8%) individuals had a registration of psychiatric morbidity before index. Of those with a history of psychiatric morbidity, 153 174 (96.7%) were exposed to affective or anxiety morbidity and 13 225 (8.3%) had a history of psychotic morbidity.

The patients in the group with psychiatric history (exposed group) were older than those with no psychiatric history (unexposed group) with a median age of 55 years (IQR 39-70) compared with 47 years (IQR 33-64). There were no differences in sex distribution with approximately 79% females in both groups.

Median follow-up time differed by approximately 6 months between the exposed and unexposed groups (63.4 vs 69.2 months) (Table 2).

**Table 2.** Characteristics of the study cohort with autoimmune hypothyroidism between 2006 and 2020

	Psychiatric history n = 158 468 (44.8%)	No psychiatric history n = 195 240 (55.2%)	*P* value
Female n (%)	125 659 (79.3)	153 620 (78.7)	<.001*^a^*
Age years median (IQR)	55 (39-70)	47 (33-64)	<.001*^b^*
Median index date	March 21, 2014	July 17, 2013	—
Affective or anxiety n (%)	153 174 (96.7)	—	—
Psychotic n (%)	13 225 (8.3)	—	—
Follow-up time months median	63.4	69.2	<.001*^b^*
Index year before 2012 n (%)	53 990 (41.4)	76 355 (58.6)	
Index year 2012 or later n (%)	104 478 (46.8)	118 885 (53.2)	<.001*^a^*

Abbreviation: IQR, interquartile range.

^*a*^Chi-square test. For index year, the test compares the distribution of psychiatric history before vs after 2012.

^*b*^Mann–Whitney U-test.

### Psychiatric Morbidity and LT3 Treatment

During the study period, a total of 12 568 (3.6%) individuals received LT3 and 341 140 (96.4%) were treated with only LT4.

The patients receiving LT3 were younger than those with LT4 monotherapy, median 43 years (IQR 34-52) vs 51 years (IQR 35-67).

Patients with a history of psychiatric morbidity prior to onset of autoimmune hypothyroidism had higher odds of receiving LT3 treatment than patients without psychiatric morbidity (aOR 1.90, 95% CI 1.83-1.97, *P* < .001). Separate analyses for each confounder revealed that age was the most influential covariate (Table 3).

**Table 3.** Associations between psychiatric history and subsequent liothyronine treatment in patients with autoimmune hypothyroidism between 2006 and 2020

Exposure	n	Incident LT3 use n (%)	OR (95% CI)	*P* value (OR)	aOR (95% CI)	*P* value (aOR)
**Primary analysis**						
Psychiatric morbidity	158 468	7182 (4.5)	1.76 (1.61-1.73)	<.01	1.90 (1.83-1.97)	<.001
No psychiatric morbidity	195 240	5386 (2.8)	—		—	
Psychiatric morbidity, age adjusted					1.91 (1.85-1.98)	<.001
Psychiatric morbidity, sex adjusted					1.67 (1.61-1.73)	<.001
Psychiatric morbidity, region adjusted					1.66 (1.60-1.72)	<.001
**Secondary analyses**						
Affective/anxiety morbidity	153 174	7034 (4.6)	1.70 (1.64-1.76)	<.01	1.91 (1.84-1.98)	<.001
No affective/anxiety morbidity	200 534	5534 (2.8)	—		—	
Psychotic morbidity	13 225	472 (3.6)	1.0 (0.91-1.10)	.92	1.08 (0.98-1.19)	.11
No psychotic morbidity	340 483	12 096 (3.6)	—		—	
**Stratified analyses**						
Psychiatric morbidity, index before 2012			1.50 (1.42-1.58)	<.01	1.80 (1.70-1.90)	<.001
Psychiatric morbidity, index 2012 or later			1.91 (1.82-2.01)	<.01	2.11 (2.00-2.21)	<.001

Abbreviations: aOR, adjusted odds ratio; LT3, liothyronine.

The strongest association with LT3 treatment was seen in the group of patients with a history of affective and anxiety morbidity (aOR 1.91, 95% CI 1.84-1.98, *P* < .001) while there was no difference in LT3 between patients with a prior psychotic morbidity compared with those without psychotic morbidity (aOR 1.08, 95% CI 0.98-1.19, *P* .11) (Table 3).

In the stratified analysis of patients with index date before and after 2012, the association between psychiatric history and LT3 use was stronger when treatment for hypothyroidism was initiated in 2012 or later (aOR 1.80, 95% CI 1.70-1.90, *P* < .001 vs aOR 2.11, 95% CI 2.00-2.21, *P* < .001) (Table 3).

## Discussion

In this retrospective nationwide register–based cohort study we found that patients with a history of psychiatric morbidity prior to the diagnosis of autoimmune hypothyroidism were nearly twice as likely to receive LT3 compared with patients without such a background. Secondary analyses showed that this association was particularly strong among patients with a history of affective or anxiety morbidity (aOR 1.91, 95% CI 1.84-1.98, *P* < .001) while no such association was observed between previous psychotic morbidity and LT3 treatment.

There are several studies indicating an association between hypothyroidism, thyroid autoimmunity, and psychiatric disease (16-19). Also, in a Danish cohort study using health registers, patients with hypothyroidism were more likely to have a premorbid history of psychiatric diagnoses and treatments, as well as an increased risk of being diagnosed and receiving psychiatric medication after the diagnosis of hypothyroidism (20). Furthermore, individuals with Type D personality, defined by heightened negative affectivity and social inhibition, have reported greater dissatisfaction with their hypothyroidism treatment and health care experience, and persistent symptoms despite thyroid hormone treatment (21).

Conversely, there are limited data on mental characteristics of patients with hypothyroidism who receive LT3. In a Scottish retrospective cohort study, the TEARS study, on adverse outcomes of LT3 use compared with LT4 only, patients prescribed LT3 were more likely to have been prescribed antipsychotic or antidepressant medication prior to starting thyroid hormone replacement (22). In a register-based cross-sectional study encompassing all Danish citizens ≥30 years with thyroid hormone therapy, treatment with LT3 was associated with higher education, and among the LT3-treated the majority were women (95%) and most commonly 40-49 years old (23).

According to current European guidelines (12), combination therapy with LT3 as an adjunct to LT4 can be considered for patients with persistent symptoms of hypothyroidism despite adequate substitution with LT4. Combination therapy is considered experimental and other underlying medical conditions explaining the symptoms such as vitamin B12 deficiency, depression, and chronic fatigue syndrome should be ruled out before LT3 initiation (12).

The increased use of LT3 among patients with autoimmune hypothyroidism and a history of psychiatric morbidity may have several explanations. Affective/anxiety disorders can persist with symptoms mimicking hypothyroidism. Individuals with these conditions who are later diagnosed with hypothyroidism may undergo more frequent assessments and testing in both psychiatric and somatic health care systems due to not feeling well and may receive adjunct medical treatments.

Another explanation might be that more patients in this group have undiagnosed hypothyroidism and because of ongoing discomfort receive treatment for depression or anxiety while worsening hypothyroidism develop, becoming refractory to standard treatments and LT3 is added as a tentative solution (24, 25).

In this study we found no increased use of LT3 in the group of patients with a history of psychotic morbidity. This may be due to a more restrictive approach to experimental treatment strategies, or a lower demand for LT3 among these patients. This result contrasts with the TEARS study, which found that both antipsychotic and antidepressant medications were more likely to be prescribed before hypothyroid treatment in LT3 users compared with LT4 users (22). However, given the relatively small number of patients with prior psychotic morbidity, the observed *P =* .11 may reflect underpowering and this finding should be interpreted cautiously.

An interesting observation is that patients receiving LT3 were younger than those treated with LT4 as monotherapy (median 43 years, IQR 34-52 and 51 years, IQR 35-67, respectively). This may be explained by younger patients being more prone to request new treatments when standard therapy fails, and older patients are less inclined to do so. Influence from media and social media on the younger population can also pose as an explanation (26). In contrast, older patients may have more comorbidities and polypharmacy that can contribute to a more restrictive approach to the use of LT3 (27).

The key strength of this study is its large, nationwide cohort comprising all patients receiving hormone substitution for hypothyroidism. The use of high-quality Swedish register data ensures reliable measurements of both exposure (psychiatric morbidity) and outcome (LT3 treatment). Temporal analyses provide insights into treatment trends. Adjusting for potential confounders, such as age, sex, and county of residence strengthen the validity of the observed associations. An important strength is the restriction to a homogeneous cohort of patients receiving thyroid hormone substitution. This reduces the heterogeneity in underlying thyroid disorders and reflects the prescription pattern in a well-defined population. However, there are several limitations. The retrospective observational design of the study limits the ability to establish causal relationships, and the associations may be influenced by residual confounding. Due to lack of data on thyroid hormone concentrations and detailed information on treatment indications, we cannot determine whether LT3 was prescribed due to laboratory abnormalities, persistent symptoms, or patients’ or treating physicians’ preferences. Also, we lack data on treatment with interferons or immune checkpoint inhibitors.

This study provides empirical evidence that patients with autoimmune hypothyroidism and a history of psychiatric morbidity are more likely to receive LT3 treatment, especially among those with affective or anxiety morbidity. However, from this study we cannot tell if LT3 treatment led to clinical improvement. The higher rate of LT3 prescription to patients with psychiatric morbidity may indicate a gap between clinical practice and current treatment guidelines. Future studies should address the clinical impact and potential effects of LT3 treatment in this subgroup of patients.

In conclusion, patients with a history of psychiatric morbidity, particularly affective or anxiety morbidity, prior to diagnosis of autoimmune hypothyroidism are more likely to receive LT3 treatment than those without such history. This may reflect lingering symptoms that influence treatment decisions of hypothyroidism, either due to symptoms being misinterpreted as treatment-resistant hypothyroidism or actual physiological differences in thyroid disease within this group.

## Data Availability

The data that support the findings of this study are available from the corresponding author upon reasonable request.

## Abbreviations

aOR: adjusted odds ratio
ATC: Anatomical Therapeutic Chemical
IQR: interquartile range
LT3: liothyronine
LT4: levothyroxine
NPDR: National Prescribed Drug Register
